# Next-Generation Adenoviral Vector-Based Vaccines for Severe Acute Respiratory Syndrome Coronavirus-2

**DOI:** 10.3390/vaccines13040406

**Published:** 2025-04-14

**Authors:** Muralimanohara S. T. Murala, Vivek Gairola, Ekramy E. Sayedahmed, Suresh K. Mittal

**Affiliations:** Department of Comparative Pathobiology, Purdue Institute of Inflammation, Immunology and Infectious Disease, College of Veterinary Medicine, Purdue University, West Lafayette, IN 47907-2027, USA; mmurala@purdue.edu (M.S.T.M.); vgairola@purdue.edu (V.G.); esayedah@purdue.edu (E.E.S.)

**Keywords:** adenoviral vector, adenoviral vector-based vaccine platform, SARS-CoV-2, SARS-CoV-2 vaccine, vaccine-induced immune thrombotic thrombocytopenia (VITT)

## Abstract

This review systematically revises adenovirus (Ad) biology, vector structure, immune responses, and currently available Ad vector COVID-19 vaccines. It analyzes the challenges associated with the Ad vector-based vaccines, including preexisting vector immunity and other side effects. Moreover, this review explores novel and innovative strategies to overcome these constraints for developing next-generation vaccines for broad protection to cover emerging SARS-CoV-2 variants. The future refinement of Ad vaccine platforms will be pivotal in achieving durable immunity against emerging variants for global preparedness.

## 1. Introduction

In late 2019, an extremely contagious coronavirus, Severe Acute Respiratory Syndrome Coronavirus-2 (SARS-CoV-2), emerged in China, causing the pandemic of acute respiratory illness known as ‘coronavirus disease 2019′ (COVID-19) [1,2]. The rapid global dissemination of SARS-CoV-2 sparked widespread public health concern and had a significant socio-economic impact worldwide. This emphasizes our vulnerability to emerging pathogens and stresses the significance of developing safe and effective vaccines to combat COVID-19. Diverse vaccine platforms, such as inactivated virus, adenoviral (Ad) vector [3], subunit [4], mRNA [5], and DNA [6], were utilized to quickly develop effective vaccines for mass production to meet the global demand. These vaccines have been pivotal in reducing the disease severity and mitigating virus spread [7,8], saving millions of lives during the COVID-19 pandemic, one of the most severe global health crises in modern history. Notably, their development was achieved in record time, with emergency use authorization granted just 11 months after the SARS-CoV-2 genome was sequenced.

Despite the significant achievements of COVID-19 vaccines, several shortcomings became apparent over time. Continuous mutations in the viral genome have led to the emergence of new variants, resulting in the reduced protective efficacy of the vaccines generated using the ancestral strains. Although these vaccines were effective in abating disease severity, they failed to prevent infection and virus transmission [9]. Furthermore, the durability of protection conferred by current COVID-19 vaccines is short [10].

Setting aside the challenges associated with vaccines, it is crucial not to underestimate the significance of Ad vector-based vaccine platforms for developing the next generation of SARS-CoV-2 vaccines with better efficacy against emerging variants. Ad vector vaccines have played a pivotal role in the global endeavor to combat SARS-CoV-2-associated illnesses and deaths [11]. Overall, Ad vector-based vaccine platforms have substantial cloning capacity, efficient transduction efficiency, ease of bulk production in a short time, are excellent in eliciting both humoral and cellular immune responses, and have a safety profile like other vaccine platforms [12]. However, one of the limitations of Ad vectors is the development of strong immune responses against the vector backbone, thereby restricting their reusability within a short duration [13]. The presence of preexisting Ad vector immunity in a high percentage of the population due to exposure to common human Ads also has the potential to curb the utility of certain human Ad vector-based vaccines [14]. Innovative strategies in vector structure, formulation, and delivery have been employed to address these limitations.

## 2. Shortcomings of Current SARS-CoV-2 Vaccines

While various vaccine platforms have been deployed to combat COVID-19, all face challenges such as waning immunity, reduced cross-variant protection, and incomplete prevention from virus transmission. Understanding these broad limitations provides context for assessing platform-specific issues, particularly those of Ad vector-based vaccines.

### 2.1. Decreased Vaccine Efficacy Against Emerging SARS-CoV-2 Variants

The existing vaccines target specific virus strains, resulting in limited cross-reactivity and reduced protection against new variants. A luminescence-based lentiviral-based pseudovirus neutralization assay demonstrated that the individuals who received two doses of the BNT162b2 or mRNA-1273 vaccines showed a robust neutralization of the wild-type virus. However, their neutralization efficacy against variants varied significantly [15]. The D614G variant showed minimal reductions in neutralization titers (1.2- to 1.4-fold decrease), while variants with RBD mutations, such as the UK B.1.1.7, Denmark B.1.1.298, and California B.1.429, exhibited moderate reductions (1.4- to 2.3-fold). More resistant strains, such as the Brazilian/Japanese P.2 and P.1 variants, showed greater decreases in neutralization titers (5.8- to 6.7-fold), and the South African B.1.351 variants exhibited the most significant reductions (27.7- to 42.4-fold). This substantial resistance to neutralization was primarily attributed to mutations in the RBD region of the S protein, although additional mutations outside the RBD were also responsible for a lower response to vaccine-induced immunity [15]. Another study in the rodent model highlighted the reduced neutralizing efficacy of Ad-vectored vaccines against emerging SARS-CoV-2 variants [16]. Similarly, Ad-vectored vaccines have shown reduced neutralization efficacy against emerging SARS-CoV-2 variants, with studies indicating that preexisting Ad immunity and antigenic drift contribute to lower immune responses against novel variants. Vaccines targeting specific variants, like Delta, provided strong neutralization against Delta variants and cross-protection against mismatched variants (e.g., Gamma), but their efficacy against Omicron subvariants (BA.2 and BA.5) significantly diminished [16], underscoring the challenge of VOCs on vaccine efficacy.

### 2.2. Short Durability of Protective Immune Responses

The duration of protection provided by different COVID-19 vaccines has shown varying degrees of decline over time based on the vaccine type and number of doses administered. For the two-dose mRNA vaccines, BNT162b2 (Pfizer–BioNTech, New York, NY, USA) and mRNA-1273 (Moderna, Cambridge, MA, USA), the effectiveness against COVID-19 infection was 94.5% and 95.9%, respectively, at 2 months post-vaccination, decreasing to 66.6% and 80.3%, respectively, by 7 months. The Ad26.COV2.S (Johnson & Johnson–Janssen, Beerse, Belgium) one-dose vaccine showed a vaccine efficacy of 74.8% at 1 month, dropping to 59.4% by 5 months [10]. For the heterologous AZD1222 and mRNA vaccine regimen, the effectiveness was 89% at 15–30 days and 66% from day 121 onwards. In contrast, the homologous AZD1222 two-dose vaccine showed a reduction in effectiveness from 68% at 15–30 days to undetectable effectiveness from day 121 onwards [17].

### 2.3. Failure to Prevent Disease Symptoms and Virus Transmission

COVID-19 vaccines have proven highly effective in reducing the severity of symptomatic disease, hospitalizations, and ICU admissions; however, their role in preventing virus transmission remains questionable [18]. A UK cohort study revealed that the transmission rates among vaccinated individuals were similar to those of the unvaccinated, thereby challenging the assumption that vaccination curbs virus spread [19]. Further studies in Israel and the USA discovered that vaccinated individuals could carry viral loads comparable to those of unvaccinated people, raising concerns about virus transmission [20]. Research on secondary attack rates (SARs) showed mixed results since no reduction in infectiousness due to vaccination was observed in a few studies, while others reported a moderate effect [21]. In addition, incorporating nucleocapsid as an antigen to improve vaccine effectiveness demonstrated that vaccines were unable to prevent breakthrough infections in K18-ACE2 mice [22]. These findings suggest that despite the benefits of COVID-19 vaccines in reducing disease severity, virus transmission from vaccinated individuals after infection remains a challenge.

## 3. Ad Biology, Vector Structure, and Immune Responses

Ads are non-enveloped dsDNA viruses classified under the family *Adenoviridae*, which comprises six genera: *Mastadenovirus*, *Aviadenovirus*, *Atadenovirus*, *Ichtadenovirus*, *Siadenovirus*, and *Testadenovirus* [23]. The predominantly used Ads for vector development include human Ads (HAds) (over eighty-five types in seven groups; A–G) [24], chimpanzee Ads (six groups; A–F) [25], bovine Ads (ten types), canine Ads (two types), porcine Ads (five types), Ovine Ads (seven types), and fowl Ads (five types) [13,26]. Ads are generally species-specific, and many of them usually cause inapparent-to-mild infections. However, in the presence of predisposing factors like stress and immunosuppression, they instigate various pathologies in humans and animals, ranging from respiratory tract infections, gastroenteritis, enteritis, keratoconjunctivitis, hepatitis, icterus, cystitis, adenitis, nephritis, polyarthritis, hydropericardium syndrome, etc. [27,28].

**Figure 1:**
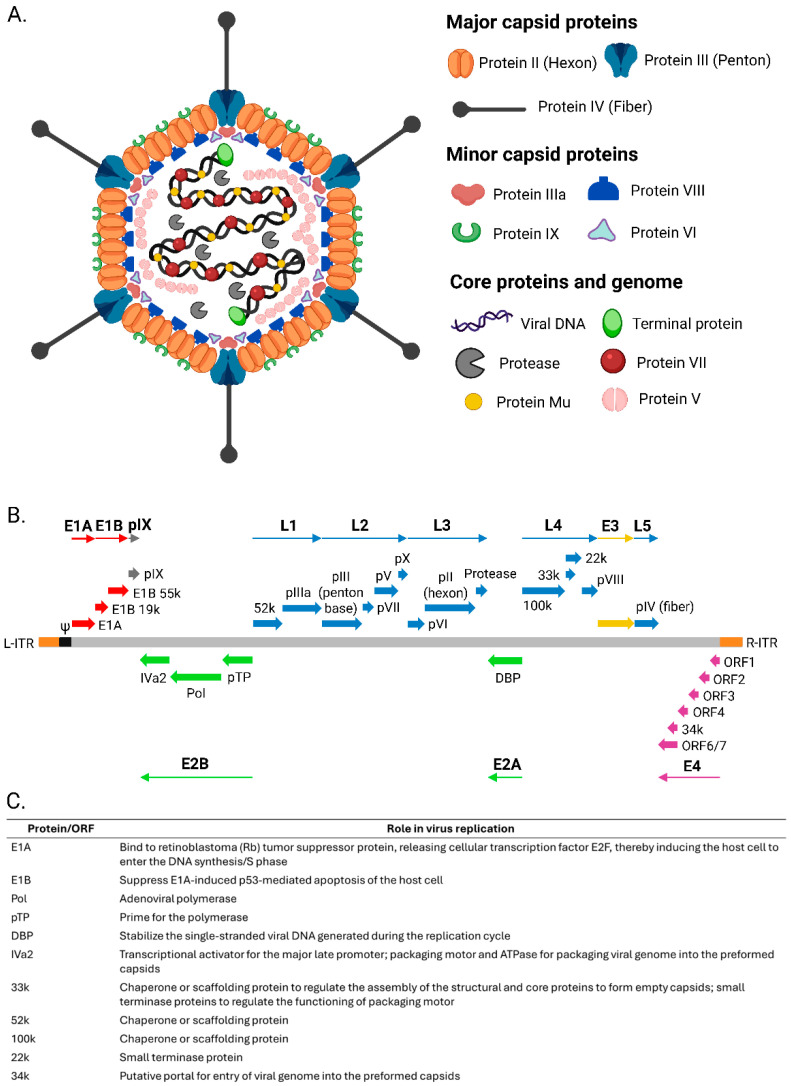
(**A**) Diagrammatic representation of adenovirus, illustrating its key proteins and genomes. (**B**) Schematic representation of the adenoviral genome, gene transcription units, and key proteins. (**C**) Overview of the adenoviral genome with annotated open reading frames (ORFs) and their functions in viral replication. E1, early region (shown as red arrows); E2 (shown as green arrows); E3 (shown as yellow arrows); E4 (shown as pink arrows); L, late region (shown as bluearrows); L’-ITR, left inverted terminal repeat; R’-ITR, right inverted terminal repeat; Ψ, packaging signal; Pol, adenovirus DNA polymerase; pTP, terminal protein; DBP, DNA-binding protein. The figure was created using https://BioRender.com.

Ad virions are 90–120 nm in size and comprise an icosahedral capsid with pseudo-triangulation number = 25 [29]. A single molecule of linear dsDNA of 24–48 kbp in size serves as the viral genome (Figure 1). It is flanked by inverted terminal repeats of 26–721 bp in length and has a covalently linked terminal protein at the 5′ end of each strand [30]. The capsid consists of 240 hexons (edges and faces) and 12 penton complexes (vertices), each consisting of the penton base and a projecting trimeric fiber (most Ads) [31]. The N-terminal stem anchors the fiber to the penton base, while the C-terminal knob serves as a ligand for the cellular receptors [31]. For most Ads, including HAds (subgroups A, C, D, E, and F), the initial high-affinity interaction of the fiber knob occurs with the coxsackie–Ad receptor (CAR; primary receptor) on the permissive host cells like epithelial cells, myoblast, endothelial cells, and hepatocytes [32]. A secondary interaction between the penton base and integrins follows, leading to cell entry [33]. CAR also serves as a primary receptor for many nonhuman Ads, including chimpanzee Ads, canine Ads, and avian Ads [34,35,36]. Other primary cell entry receptors used by Ads include CD80/86 and CD46 (subgroup B HAds), α (2,3)-linked and α (2,6)-linked sialic acid (BAds), heparan sulfate proteoglycans (subgroup B HAds), polysialic acid (HAd52), vascular cell adhesion molecule-1 (HAd52), and desmoglein-2 (subgroup B HAds; HAd3,7,11, and 14) [37,38,39].

Ad attachment triggers a signaling cascade, and the virus ultimately enters the cell via clathrin-coated endosomes [40]. In the endosome, the capsid is partly disassembled, the endosomal membrane disintegrates, and the virion escapes into the cytoplasm [41]. The virion is then carried to the nuclear pore complex (NPC) by dynein microtubules, and the viral genome, along with the core proteins, are subsequently delivered to the nucleus [42,43]. The viral genome is transcribed as five early (E1A, E1B, E2, E3, and E4), two intermediate (IX and IVa2), and one late (L) transcriptional unit. The early genes’ products make host cells conducive for virus replication and modulate host immune responses [44]. Intermediate and late gene products take part in viral assembly and maturation [44]. They encode structural proteins, i.e., capsomers and core proteins, which assemble to form empty capsids under the regulation of chaperons or scaffolding proteins [45,46]. The packaging proteins consisting of the packaging motor (IVa2) and small terminates (22K and 33K) interact with cis-acting packaging positioned next to the left ITR and package the viral genome into the empty capsids [47,48]. Mature virions are subsequently released from the host cell following lysis.

**Figure 2:**
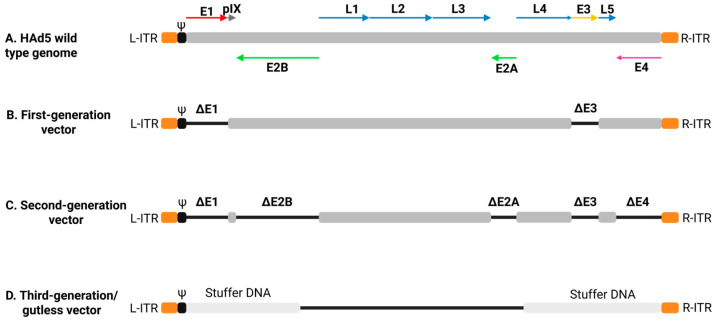
Schematic representation of adenoviral vector generations. (**A**) Wild-type adenovirus genome. (**B**) First-generation adenoviral vector with deletions in the E1 and E3 genes. (**C**) Second-generation adenoviral vector with all early genes (E1, E2, E3, and E4) removed. (**D**) Third-generation adenoviral vector, retaining only the inverted terminal repeat (ITR) regions while the rest of the genome is deleted. The packaging signal (Ψ) is located immediately downstream of the ITR sequence. The figure was created using https://BioRender.com.

Several Ads have been developed for vectored vaccines and gene therapy [49,50]. Ad vector design strategies have evolved to enhance ease in vector recovery, safety, and transgene expression and to modulate innate immune responses (Figure 2A). Different Ad types exhibit varying levels of virulence, with some causing mild respiratory or enteric disease, while others lead to systemic infections. To mitigate potential risks, attenuation strategies such as gene deletions and modifications have been employed to enhance vector safety. There are three generations of vectors based on Ad gene deletion regions [51]. First-generation Ad vectors lack either E1 or both E1 and E3 regions, rendering the vector replication incompetent and increasing the foreign gene insertion capacity (Figure 2B) [52]. However, these vectors can only proliferate in a cell line, like the human embryonic kidney cell line 293 (HEK-293), which continuously expresses E1 proteins [53]. Second-generation Ad vectors were engineered by deleting additional gene regions such as E2 and/or E4, along with E1 and E3, aiming to diminish vector immunogenicity and increase the transgene insertion capacity (Figure 2C) [54]. Third-generation Ad vectors or gutless Ad vectors are devoid of all Ad genes, excluding ITRs and genome packaging sequences, resulting in enhanced safety and reduced immunogenicity to the vector, but their production requires a helper Ad (Figure 2D) [55,56,57].

The adjuvant action of Ad vectors results in enhanced innate immune responses, leading to robust adaptive immune responses [58]. Ad pathogen-associated molecular patterns (PAMPs) interact with multiple endosomal and cell surface pathogen recognition receptors (PRRs) on antigen-presenting cells (dendritic cells and macrophages), culminating in a cascade of immune signaling [59,60]. Following attachment to host cells, viral surface proteins interact with the cellular receptors (e.g., CAR and integrins) and activate Toll-like receptors (TLRs), including TLR2, TLR3, TLR4, TLR7, and TLR9 [58]. TLR-independent cytosolic DNA sensors like cyclic GMP-AMP synthase/stimulator of interferon genes (cGAS/STING) and NLR family pyrin domain-containing 3 (NLRP3) inflammasomes also become activated [61,62].

HAd5 is the most widely used Ad vector in vaccine research due to its well-characterized genome, high transduction efficiency, and ability to elicit both humoral- and cell-mediated immune responses [63,64,65,66]. The HAd5 hexon protein also interacts with the blood coagulation factor X (FX) and activates TLR4 [67]. Various adaptor proteins, like myeloid differentiation primary response 88 (MyD88), interferon regulatory factor 3 (IRF3), TNF receptor-associated factor 6 (TRAF6), and TIR domain-containing adaptor-inducing interferon (TRIF), mediate the downstream signaling of these pathways and ultimately activate nuclear factor-kappa B (NF-κB) [68,69]. Upregulated NF-κB boosts the expression of various proinflammatory cytokines and chemokines, including interferon-gamma (IFN-γ), interleukin (IL)-1, tumor necrosis factor-alpha (TNF-α), IL-1β, IL2, IL8, IL6, macrophage inflammatory protein alpha (MIP-α), MIP-1, MIP-2, C–C chemokine ligand 2 (CCL2), CCL3, CCL4, C-X-C motif chemokine ligand 1 (CXCL1), CXCL2, and others [70,71,72].

Innate immune responses induced by Ad initiate the adaptive immune responses targeted toward the transgene and vector proteins. The expressed antigens are presented to naïve T cells, which subsequently differentiate into effector and memory cells [73]. However, the induction of vector-specific neutralizing antibodies and vector-specific CD4+ and CD8+ T cells wanes vector efficacy, thereby significantly attenuating the expression of the transgene following the re-administration of the same vector [12].

## 4. Licensed Ad Vector-Based SARS-CoV-2 Vaccines

At the start of the SARS-CoV-2 pandemic, efforts were made to develop Ad vector-based COVID-19 vaccines utilizing human Ad (HAd5 and HAd26) and chimpanzee Ad (ChAd) platforms containing the genetically modified SARS-CoV-2 spike (S) gene [3]. These Ad-vectored vaccines expressed high levels of the S protein, inducing robust S-specific humoral and CMI responses following vaccination [74,75]. Ad-vectored COVID-19 vaccines served a vital role in combating COVID-19-related sickness and deaths worldwide (Table 1).

### 4.1. AZD1222 Vaccine

AZD1222, a ChAd (ChAdOx1) vector expressing the SARS-CoV-2 S protein, was developed by AstraZeneca in collaboration with the University of Oxford, UK [76]. The Serum Institute of India has developed its version of AZD1222, known as Covishield [77].

The preclinical evaluation of the AZD1222 vaccine in rhesus macaque monkeys (six animals/group) using a one- or two-dose regimen of 2.5 × 10^10^ virus particles (VPs) administered intramuscularly was pursued [78]. The vaccine showed no adverse events. S-specific neutralizing antibodies appeared within 14 days post-vaccination, with a notable increase following the booster dose. The vaccinated monkeys exhibited lower clinical disease scores and reduced lung viral loads following the challenge with SARS-CoV-2 compared to the control group.

The clinical safety, efficacy, and immunogenicity of the AZD1222 vaccine were further investigated in five key clinical trials: COV001 (UK, Phase 1/2), COV002 (UK, Phase 2/3), COV003 (Brazil, Phase 3), COV004 (Kenya, Phase 1/2), and COV005 (South Africa, Phase 1/2). In COV001, out of 1077 participants, 543 received a standard dose (SD) of AZD1222 (5 × 10^10^ VP), while the others received a control vaccine. After one dose, S-binding and virus-neutralizing antibodies were detected, with a significant increase in post-boost given 56 days later in a small segment of participants. The immune response was Th1 biased, characterized by an increased production of IFNγ and TNFα [74]. The COV002 trial involved 560 participants divided into three age groups: 18–55, 56–69, and ≥70 years. Two dose regimens were tested: a low dose (LD) (2.2 × 10^10^ VP) and an SD (3.5–6.5 × 10^10^ VP). The antibody responses were consistent across all age groups, though slightly lower in the LD group. The vaccine demonstrated better tolerability in older adults. In this trial, AZD1222 achieved an efficacy of 70.4% with two doses. Efficacy increased to 90.0% when the first dose was low, and the second dose was standard. Higher efficacy was observed with longer intervals between doses, with a median interval of 69 days in the SD/SD group [79].

The COV003 trial focused on high-risk participants, including healthcare workers, across six sites in Brazil. Participants received two SDs of AZD1222 (3.5–6.5 × 10^10^ VP) administered up to 12 weeks apart. The vaccine efficacy in this trial was 62.1% [80]. The COV004 study was a Phase 1/2 randomized controlled trial conducted in Kenya to evaluate the safety and immunogenicity of AZD1222 compared to a rabies vaccine. A total of 400 volunteers participated, with 200 receiving AZD1222 and 200 receiving the rabies vaccine. Results showed that 28 days after the second dose, 99.5% of participants vaccinated with AZD1222 developed anti-S IgG antibodies, compared to 41.2% in the rabies vaccine group [81]. The COV005 trial was conducted at seven sites in South Africa and included adults with and without HIV, who received two doses (3.5–6.5 × 10^10^ VP) of AZD1222 or a placebo, with dose intervals ranging from less than 4 weeks to a maximum of 12 weeks. Vaccine efficacy against symptomatic and asymptomatic infections was 90.6% for wild type, 77.1% for Delta, and 6.7% for Beta variants [82].

A large, randomized, double-blind, placebo-controlled Phase 3 trial carried out in the United States, Chile, and Peru further evaluated the safety, efficacy, and immunogenicity of two doses (5 × 10^10^ VP) of AZD1222 in 32,451 participants, including older adults. AZD1222 was safe, with incidences of serious adverse events being low and comparable to those of the placebo group. Most local and systemic reactions were mild to moderate. The overall vaccine efficacy against symptomatic COVID-19 was estimated at 74%. In participants aged 65 years or older, the vaccine efficacy was 83.5%. Severe or critical COVID-19 cases were not observed among fully vaccinated participants, while eight cases occurred in the placebo group. The vaccine efficacy for preventing SARS-CoV-2 infection was 64.3% [80].

### 4.2. Ad26.COV2.S Vaccine

The HAd26 vector-based COVID-19 vaccine expressing a modified version of the S protein of SARS-CoV-2, Ad26.COV2.S, was developed by the Janssen Pharmaceutical Company of Johnson & Johnson [3]. The S gene modifications included the replacement of amino acids at positions 986 and 987 with two prolines (2Ps) to stabilize the S protein in a pre-fusion conformation. The preclinical studies with a single dose of the Ad26.COV2.S vaccine demonstrated strong humoral and CMI responses and protection efficacy across various species, including mice, rabbits, Syrian hamsters, and nonhuman primates [75,83]. The vaccine significantly reduced the lung viral load following a SARS-CoV-2 challenge in Syrian hamsters, while nonhuman primates showed undetectable viral loads in both the nose and lungs. Six months post-vaccination, most macaques maintained undetectable lung viral loads, indicating long-lasting protection [83,84,85].

The Ad26.COV2.S vaccine was evaluated clinically through five key trials assessing safety, immunogenicity, and efficacy. COV1001 was a multicenter, randomized, placebo-controlled Phase 1–2a study that involved healthy adults aged 18–55 and those 65 years or older. Participants received a 5 × 10^10^ VP (LD) or 1 × 10^11^ VP (high dose) of the vaccine in a two-dose or single-dose regimen. Adverse events were mild to moderate and commonly included fatigue, myalgia, headache, and pain at the injection site, while fever was less common. The adverse reactions were lower in older participants and those receiving the LD. Neutralizing antibody responses were robust, with seroconversion rates of 90% or higher by day 29, and T-cell responses showed a skew toward Th1 helper T cells. The second dose significantly boosted antibody titers [86]. COV1002 was conducted in Japan, involving healthy adults aged 20–55 and 65 years or older who received two doses of Ad26.COV2.S 56 days apart. Neutralizing antibody responses observed at 29 days post-vaccination were comparable to those in COV1001 [87]. COV2001, a randomized, placebo-controlled Phase 2a trial, was conducted in Germany, Spain, and the Netherlands. The study involved adults aged 18–55 and those 65 years or older. It evaluated the vaccine’s safety, reactogenicity, and humoral immune response. Participants received higher doses of Ad26.COV2.S, which were found to be more immunogenic and reactogenic than lower doses. Neutralizing antibodies (VNAs) and S-binding antibodies (S-bAbs) were observed to persist for at least six months following a single 5 × 10^10^ VP dose. Longer intervals between doses were shown to enhance immune responses, suggesting robust immune memory [88]. COV3001, a pivotal Phase 3 trial, evaluated the single-dose regimen of Ad26.COV2.S. The trial enrolled 39,321 SARS-CoV-2-negative participants, who were randomized 1:1 to receive either Ad26.COV2.S (5 × 10^10^ VP) or the placebo. The vaccine demonstrated an efficacy of 66.9% at ≥14 days and 66.1% at ≥28 days against moderate-to-severe COVID-19 infection. Protection against severe-to-critical cases was 76.7% at ≥14 days and 85.4% at ≥28 days. In South Africa, where the 20H/501Y.V2 variant was predominant, vaccine efficacy ranged from 52% to 64% against moderate-to-severe cases and reached 81.7% for severe cases [89]. COV3009, a Phase 3 trial, evaluated a two-dose regimen with a booster dose at 2 months and enrolled 31,300 participants across 10 countries. Vaccine efficacy against moderate-to-severe COVID-19 was 75.2% (95% CI 54.6–87.3) at least 14 days post-booster, primarily against the alpha and mu variants. Safety analysis in 6067 participants indicated transient, mild-to-moderate adverse events with similar frequencies after primary and booster doses [90].

### 4.3. Gam-COVID-Vac Vaccine

Gamaleya Research Institute, Russia, developed Sputnik V (Gam-COVID-Vac), employing HAd26 and HAd5 vectors expressing the S protein of SARS-CoV-2 for the prime and boost approach [91]. Preclinical studies were conducted in several animal models to assess the systemic toxicity, immunotoxicity, and allergenicity of Gam-COVID-Vac. Single-dose toxicity studies in mice, rabbits, and primates were pursued with higher doses than those intended for human use. Allergenicity was tested in guinea pigs, and immunotoxicity was evaluated in mice. The results indicated no signs of toxicity, allergenicity, or immunotoxicity. However, these studies have not been published as peer-reviewed articles [91].

In Phase I, nine volunteers received components 1 or 2 of Gam-COVID-Vac and were monitored for 28 days. In Phase II, 20 volunteers were primed with component 1, and at 21 days post-prime, boosted with component 2, and then monitored until day 42. The results from Phase I inferred that both components were safe and immunogenic. Phase II demonstrated a significant increase in humoral immunity, including S-specific and VNAs. In addition, there was a marked increase in CD4/CD8 lymphocyte proliferation and IFN-γ secretion in immunized individuals [92].

A randomized, double-blind, placebo-controlled Phase 3 trial enrolled participants aged 18 and older who were negative for SARS-CoV-2 without a history of recent infections or vaccinations. The trial was conducted at 25 sites in Moscow, Russia, and participants were assigned randomly in a 3:1 ratio to receive either the vaccine or a placebo. Sixteen COVID-19 cases were reported among 18,695 participants who received both doses of the vaccine compared to 62 cases in the placebo group, resulting in a vaccine efficacy of 91.6%. The vaccine provided 100% protection against moderate-to-severe COVID-19, with nearly the same efficacy across age groups or genders. The seroconversion rate of 98.64% was achieved in vaccinated subjects by day 42, indicating the development of strong humoral and CMI responses [93].

### 4.4. Convidecia Vaccine

The Convidecia (Ad5-nCOV) vaccine, developed by CanSino Biologics Inc., Tianjin, China, was based on HAd5 expressing the SARS-CoV-2 S protein [94]. Preclinical studies with the vaccine demonstrated strong immunogenicity and protection efficacy in animal models, including mice, rats, guinea pigs, ferrets, and cynomolgus monkeys. A dose–response effect in inducing S-specific humoral and CMI responses was observed in mice, guinea pigs, rats, and cynomolgus monkeys, and protection studies were conducted in hACE2 transgenic mice, ferrets, and rhesus monkeys [95,96].

The Phase 1 clinical study of the Ad5-nCoV vaccine was conducted in Wuhan, China, in 108 adults aged 18–60, who received one of three doses (5 × 10^10^, 1 × 10^11^, or 1.5 × 10^11^ VP). All dose groups showed good safety and immunogenicity [14]. The Phase 2 study was conducted in 508 participants immunized with the low (5 × 10^10^ VP) or medium (1 × 10^11^ VP) dose. Binding antibodies were elicited in at least 94%, including VNAs in at least 75% of the participants on day 28 [97]. The Phase 3 trial was pursued in 36,982 participants from Argentina, Mexico, Chile, Pakistan, and Russia, and the Ad5-nCoV vaccine elicited significantly higher levels of S- or RBD-specific IgG antibodies and VNAs. There was a 32-fold increase in geometric mean antibody titers, with seroconversion rates of 92% for RBD-specific IgG antibodies and 76% for VNAs [98].

The AZD1222 and Ad26.COV2-S vaccines were developed using a nonhuman Ad (ChAdOx1) or less prevalent HAd26, respectively, to overcome preexisting Ad vector immunity. Similarly, the Sputnik V vaccine employed a prime and boost approach utilizing HAd26 and HAd5 with the objectives of mitigating preexisting Ad vector immunity and inducing higher levels of S-specific immune responses. The use of billions of Ad vector-based vaccine doses in humans led to protection against SARS-CoV-2-associated disease and death.

**Table 1:** Comparison of adenoviral vector-based COVID-19 vaccines.

Vaccine	Vector Type	Antigen Design	Dosing Regimen	Storage Conditions	Variant Efficacy	References
ASTRAZENECA (AZD1222)	ChAd (ChAdOx1)	Full-length spike protein with stabilizing changes	Two doses, i.m.	−20 °C for long-term storage; 2–8 °C for short-term storage	Effective against B.1.1.7; reduced efficacy against B.1.351	[82,99,100]
JANSSEN (Ad26.COV2.S)	HAd26	Full-length spike protein with stabilizing mutations	Single dose, i.m.	2–8 °C for up to 3 months; −20 °C for up to 2 years	Effective against B.1.1.7, B.1.351, and P.2 variants	[89,101]
SPUTNIK V (Gam-COVID-Vac)	HAd26 for prime, HAd5 for boost	Full-length spike protein using prime-boost strategy	Two doses, i.m.	Available in two formulations: frozen (−18 °C) and lyophilized (2–8 °C)	Effective against B.1.1.7, but neutralizing antibody response is significantly reduced against B.1.351, P.1, and B.1.1.28 variants	[102,103]
CONVIDECIA (Ad5-nCoV)	HAd5	Full-length spike protein	Single dose, i.m.	Stored at 2–8 °C	Limited data available regarding efficacy against emerging variants	[94,97,104,105]

## 5. Challenges Associated with Ad Vector-Based Vaccines

Certain side effects or challenges are expected with any vaccine platform. Potential challenges associated with Ad vector-based vaccines include preexisting Ad immunity, vaccine-induced thrombotic thrombocytopenia, Guillain–Barré syndrome, and limited protection efficacy against variant strains. In this section, some of the potential challenges are discussed.

### 5.1. Preexisting Ad Immunity

**Figure 3:**
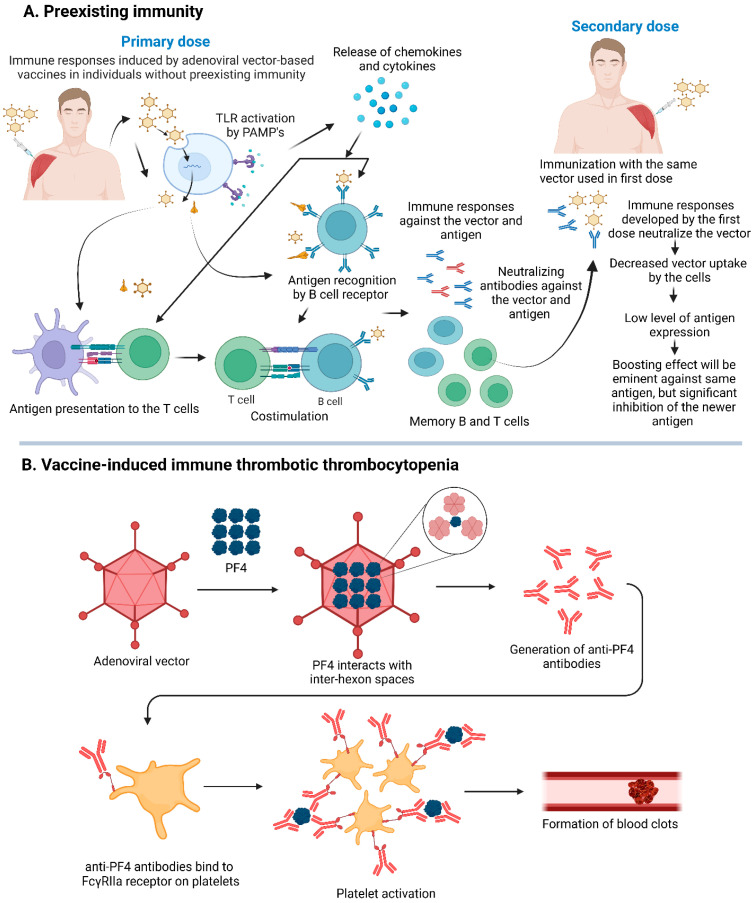
Challenges associated with adenoviral vector-based vaccines. (**A**) Immune responses in individuals to adenoviral vector vaccines following the first and second inoculation. (**B**) Potential mechanism of vaccine-induced thrombotic thrombocytopenia. TLR, Toll-like receptor; PAMP, pathogen-associated molecular patterns; PF4, platelet factor 4. The figure was created using https://BioRender.com.

There are over 85 types of HAds that infect humans, leading to infections ranging from inapparent to severe disease [106]. Due to their high prevalence, individuals are exposed to one or more types of HAds during their childhood and continue to obtain repeated infections later in life. In addition, the immunization of people with Ad-vectored COVID-19 vaccines used during the SARS-CoV-2 pandemic also contributed toward preexisting Ad vector immunity [75,76,91,96]. Sero-surveillance studies have elucidated that 40–90% of individuals harbor VNAs against HAd5, the most extensively studied HAd [106,107]. Preexisting Ad immunity, arising from prior natural Ad infections and vaccination, affects the efficacy of Ad-vectored vaccines by hindering vector uptake and subsequently reducing the development of immunogen-specific adaptive immune responses [108] (Figure 3A). Individuals with preexisting HAd5 immunity showed reduced levels of immunogen-specific antibody responses following immunization with the HAd5-based COVID-19 vaccine compared to those with minimal preexisting HAd5 immunity [97]. A substantial preexisting HAd5 neutralizing antibody titer (>1:200) significantly diminishes the neutralizing antibody titers against SARS-CoV-2 in individuals immunized either by the intranasal or intramuscular route [97]. Additionally, there were reductions in the number of IFN-γ-secreting cells in response to the SARS-CoV-2 spike protein [12,97]. Moreover, in sub-Saharan Africa, approximately 40% of human serum samples show the presence of VNAs against ChAd6, while about 15% show VNAs against ChAd7. This suggests that vaccines based on ChAd6 may have lower efficacy in the region [109].

To investigate the persistence of Ad vector immunity and its influence on the generation of adaptive immune responses against the vaccine immunogen, naïve or HAd5-primed mouse groups were vaccinated with HAd-H5HA [HAd expressing hemagglutinin (HA) of the H5N1 influenza virus] at 1, 3, 6, and 10 months post-HAd5 priming. H5HA-specific immune responses and protective efficacy were monitored [110]. A sufficient decline in HAd5 immunity was observed six months after HAd5 priming, allowing successful protective immunity using the same vector system. This suggests that annual vaccination using the same vector may be feasible due to the significant decline in vector immunity.

### 5.2. Vaccine-Induced Immune Thrombotic Thrombocytopenia

One of the rare but serious side effects of vaccines is vaccine-induced immune thrombotic thrombocytopenia (VITT), characterized by the formation of blood clots and a decrease in platelet levels after vaccination [111]. VITT and heparin-induced thrombocytopenia (HIT) share similarities in their pathophysiology [112]. For VITT, it has been proposed that the positively charged platelet factor 4 (PF4) protein interacts with the negatively charged inter-hexon spaces of Ad [113]. This interaction creates a platform for subsequent events, including the formation of antibodies against a complex formed by the binding of PF4 to the inter-hexon spaces. These anti-PF4 antibodies then bind to the platelets’ FcγRIIa receptor via their Fc region, leading to the antibody-mediated activation of platelets and the formation of blood clots, especially in cerebral veins (cerebral venous sinus thrombosis) and splanchnic circulation [114,115] (Figure 3B). Similarly, in HIT, platelet activation is triggered by antibodies formed against PF4 when it binds to a substrate, highlighting a shared mechanism in both conditions [111]. While the two conditions share similarities, it is important to note that they have distinct triggers (heparin in HIT and vaccines in VITT). In VITT, anti-PF4 antibodies seem to exhibit greater persistence compared to HIT. Antibodies to the PF4 complex in VITT can continue to stimulate platelet activation for many months after the initial acute phase and are detected for nine months or beyond [116,117,118]. In contrast, antibodies to the PF4 complex have half-lives ranging from 50 to 85 days for HIT [119]. In two studies on VITT, the interval between vaccination and the onset of VITT symptoms was observed after nine days post-Ad26.COV2.S vaccination or fourteen days post-AZD1222 vaccination [120]. Clinical symptoms in patients were chronic headache or pain in the abdomen, sporadic breathing, high blood pressure, discomfort in the chest, limb swelling, or serious cardiopulmonary difficulties. The classical cases showed both blood clot formation and a decrease in platelet levels [111]. It may be advisable not to use Ad vector-based vaccines in people with a history of blood clotting abnormalities.

The incidence of VITT varies significantly across different COVID-19 vaccines. For the AZD1222 vaccine, VITT rates range from 3.2 to 16.1 cases per million doses [121], while for the Ad26.COV2.S vaccine, the incidence is between 1.7 and 3.7 cases per million doses [122]. The Ad5-nCoV vaccine has a notably lower incidence, with only 0.0081 cases per million doses [123]. No VITT cases had been reported following the administration of the Sputnik V vaccine in Russia. However, data from the Argentinian Ministry of Health reported 13 VITT cases, including 11 cases linked to AZD1222 (0.37 cases per million doses), and 2 cases after the administration of over 20 million doses of Sputnik V [123]. The European Medicines Agency has attributed 98.5% of VITT cases to Ad-vectored vaccines, with AZD1222 and Ad26.COV2.S responsible for 90% and 8.5% of cases, respectively [124]. While VITT is extremely rare following mRNA vaccines such as BNT162b2 and mRNA-1273 [120,125], no confirmed VITT cases have been associated with inactivated whole-virion vaccines like CoronaVac and BBV152. However, two VITT cases have been reported with BBIBP-CorV, an inactivated vaccine by Sinopharm [126]. VITT can develop after the first or second vaccine dose, but most cases arise after the initial dose [127]. It has been reported that the average mortality rate for VITT was about 32%, determined by a meta-analysis of 18 studies conducted in 2021 [128,129].

### 5.3. Guillain–Barré Syndrome

Guillain–Barré syndrome (GBS) is a rare neurological disorder where the immune system targets the peripheral nerves, leading to nerve weakness, sensory irregularities, and autonomic nervous system dysfunction. This happens due to the damage to both peripheral nerves and nerve roots [130]. This condition is likely indicative of an autoimmune disorder arising from molecular mimicry [131]. There are two hypotheses for the elevated risk of GBS following vaccination with Ad vector-based vaccines. The first hypothesis suggests that the production of vector-specific antibodies may cross-react with proteins associated with myelin and axonal functions, contributing to GBS [132]. The second hypothesis assumes that Ad vectors may invade the peripheral nervous system, initiate inflammation, and subsequently lead to neuropathies [132]. The incidence of GBS following COVID-19 vaccination varies depending on the type of vaccine, with the majority of cases associated with the AstraZeneca (AZD1222) vaccine, with 3.8 excess cases per million doses in England [133]. Similarly, in Kerala, India, GBS cases after AstraZeneca vaccination were 1.4- to 10-fold higher than expected [134], and in Australia, the frequency was reported to be 1.6 times higher than usual [135]. The AstraZeneca vaccine was responsible for 56% of the recorded cases, followed by the Pfizer (20%), J&J (7%), Sputnik (7%), Moderna (5%), Sinovac (4%), and Novavax (1%) vaccines, suggesting that all COVID-19 vaccines were linked to GBS. GBS primarily affected males, accounting for 59.4% of cases, with the highest incidence in individuals aged 41–60 [136].

## 6. Strategies for Developing Next-Generation Ad Vector-Based SARS-CoV-2 Vaccines

### 6.1. Targeting Other SARS-CoV-2 Immunogens in Addition to S

**Figure 4:**
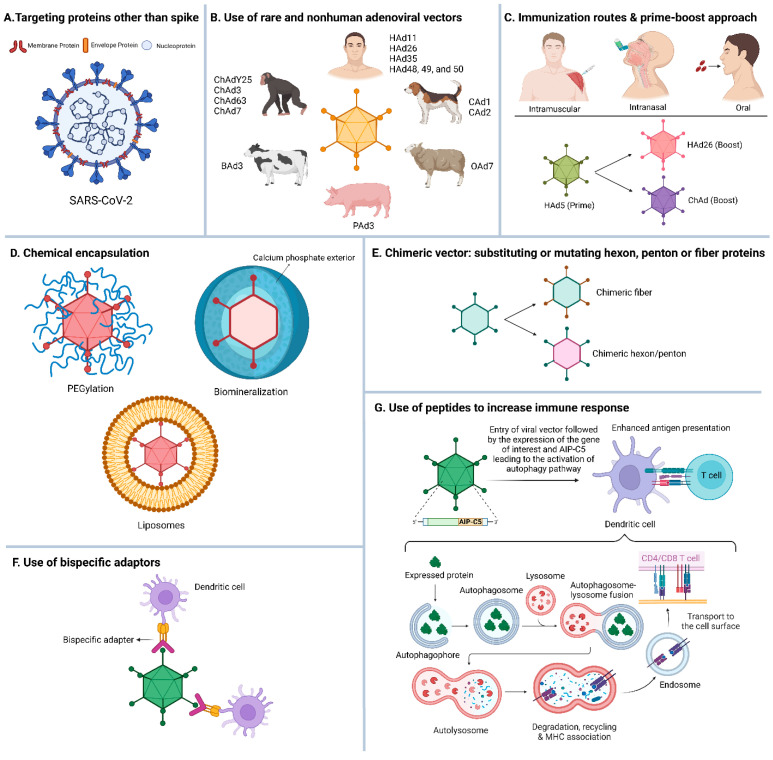
Strategies for developing next-generation adenoviral vector vaccines for SARS-CoV-2. (**A**) Use of rare and nonhuman adenoviruses as vectors. (**B**) Varied immunization routes and prime-boost approach. (**C**) Targeting proteins other than spike. (**D**) Chemical encapsulation. (**E**) Use of bispecific adapters. (**F**) Use of chimeric vector. (**G**) Use of peptides to increase the immune response. HAd, human adenovirus; ChAd, chimpanzee adenovirus; BAd, bovine adenovirus; PAd, porcine adenovirus; OAd, ovine adenovirus; CAd, canine adenovirus; AIP, autophagy-inducing peptide C5. The figure was created using https://BioRender.com.

Most Ad vector-based COVID-19 vaccines induced SARS-CoV-2 S-specific immune responses. For developing the next-generation SARS-CoV-2 vaccines that could elicit broader protection against emerging variants, it may be critical to supplement the S protein with other relatively conserved immunogens of SARS-CoV-2 such as membrane (M), envelope (E), and nucleocapsid (N) [137] (Figure 4A). The immunization of mice with DNA vaccines encoding M and E proteins provided enhanced protection following challenge with SARS-CoV-2 compared to the groups vaccinated with DNA vaccines encoding either M or E proteins [138], suggesting that including additional immunogens in the vaccine formulation is vital for broader protection. An Ad vector expressing both N and S antigens conferred reduced viral titers in both the lungs and brain, whereas the vector expressing only S led to reduced viral titers only in the lungs [22].

### 6.2. Bivalent or Multivalent S-Based Vaccine Approach

Bivalent S-based vaccines contain the S protein from both the ancestral SARS-CoV-2 and one of the recent variants, like Omicron, aiming to elicit immune responses relevant to the circulating strains [137]. Individuals having S-specific memory B and T cell responses due to vaccination with a monovalent S-based vaccine or SARS-CoV-2 exposure were immunized with a bivalent vaccine formulation, resulting in increased levels of immune responses against ancestral and new variants [139,140]. In addition, it is anticipated that new epitopes of the S protein of the recent variant will elicit S-specific immune responses against the recent strain, leading to stronger protective efficacy against the circulating strains. A ChAd vector-based bivalent vaccine expressing the S proteins of two variants elicited VNAs, serum IgA, and IgG against the ancestral and Omicron strains and conferred protection against the D614G (WA1/2020) variant and Omicron variants (XBB.1.5 and BQ.1.1) in K18-hACE2 transgenic mice and hamsters [141]. Similarly, the chimeric HAd5/35 vaccine platform expressing the S protein of BA.5 and BA.2.75 (bivalent) or the S protein of XBB/BN.1/BQ.1.1 or XBB.1.5/BN.1/BQ.1.1 (trivalent) demonstrated significantly improved cross-neutralization compared to their monovalent counterparts in mice and macaques, suggesting that multivalent approaches are more effective in generating VNAs against a wide range of circulating Omicron subvariants, offering broader protection against SARS-CoV-2 [142]. Moreover, a tetravalent recombinant protein vaccine (SCTV01E) formulated with the S ectodomains of Alpha, Beta, Delta, and Omicron BA.1 variants developed cross-neutralizing immunity against various SARS-CoV-2 strains [143]. In the Phase 3 trial, participants previously vaccinated with BBIBP-CorV (inactivated SARS-CoV-2 vaccine) were administered a booster dose of either BBIBP-CorV or SCTV01E, and the group receiving the booster dose with SCTV01E elicited significantly higher neutralizing antibody titers against the Delta and Omicron (BA.1 and BA.5) variants compared to the BBIBP-CorV group, indicating enhanced cross-variant protection due to the multi-antigen strategy [144].

### 6.3. Use of Less Prevalent Human or Nonhuman Ads as Vaccine Vectors

Different strategies have been developed to address the issues related to preexisting Ad vector immunity, and one of the approaches involves utilizing uncommon HAd types as delivery vectors. HAd11, 26, 35, 48, 49, and 50 are less prevalent in the human population, and thus are less likely to be impacted by preexisting Ad vector immunity [145,146,147]. HAd35 and HAd26 vaccine vectors targeting Ebola, HIV, malaria, tuberculosis, and COVID-19 elicited high levels of antigen-specific humoral and cellular immune responses in humans [3,148,149,150,151,152]. However, Ebola vaccines based on HAd35 or HAd26 exhibited lower immunogenicity and protection against the Ebola virus in nonhuman primates compared to the HAd5-vectored vaccine [108]. This finding underscores that seroprevalence alone does not determine vector potency factors, such as transgene expression efficiency, dendritic cell targeting, and innate immune activation. However, the vectors based on rare Ad types have demonstrated strong efficacy in prime-boost combinations. In nonhuman primates, HAd26/HAd35 heterologous regimens provided uniform protection against Ebola, with HAd26 showing a clear dose–response relationship and outperforming HAd35 in antibody titers [108].

Moreover, various nonhuman Ads derived from chimpanzee, canine, bovine, ovine, porcine, avian, and others have been engineered as vaccine vector platforms [153] to complement or substitute HAds to circumvent preexisting Ad immunity (Figure 4B). ChAd vectors offer advantages for vaccine development since they can be cultured in certified HEK293 cells with high titers [25]. There is a relatively low prevalence of ChAd cross-VNAs due to preexisting Ad immunity in the human population [154]; therefore, ChAd vector-based vaccines induce robust humoral and CMI responses even in the presence of HAd5 immunity [155,156,157].

Various ChAd vectors expressing diverse immunogens of Ebola virus, HIV, malaria, SARS-CoV-2, and respiratory syncytial virus resulted in antigen-specific immune responses [158,159]. ChAd63, a low seroprevalence ChAd, expressing a fusion protein (ME-TRAP) consisting of a string of multi-epitope (ME) fused to the *Plasmodium falciparum* pre-erythrocytic thrombospondin-related adhesion protein (TRAP) produced robust immune responses and protection against malaria in nonhuman primates [160]. ChAd3 expressing a distinctive target immunogen (NSmut), derived from conserved sequences spanning the nonstructural genes (NS3-NS5B) of hepatitis C virus genotypes 1b and BK, and the glycoprotein of Ebola virus, elicited excellent antigen-specific immune responses [161]. ChAdOx1, based on ChAd Y25-type vectors expressing immunogens of SARS-CoV-2, influenza, or *Mycobacterium tuberculosis*, led to antigen-specific robust immune responses [78,82,162,163,164]. The COVID-19 vaccine, ChAdOx1-nCoV-19, is described earlier in this review.

Humans do not have bovine Ad type 3 (BAd3) cross-VNAs [165], HAd5 antibodies do not cross-neutralize BAd3 [166], BAd3 uses α-2,3- and α-2,6-linked sialic acid receptors for virus entry [37], the virus exhibits distinct tissue tropism [167], and it efficiently transduces various human and nonhuman cell types [37]. BAd vectors induce a robust activation of innate immunity [13], leading to enhanced antigen-specific immune responses and protection better than that of HAd5 vectors [168]. Therefore, the BAd3 vector system offers an attractive vaccine platform capable of evading preexisting Ad immunity. CAd2 vectors showed promising results in immunogenicity and protection studies for rabies, rabbit hemorrhagic disease, foot-and-mouth disease, and toxoplasmosis [145,169,170].

### 6.4. Different Immunization Routes for Prime and Boost with the Same Ad Vector-Based Vaccine

The second inoculation with the same Ad vector-based vaccine results in noticeable inhibition in the immunogen expression due to the vector immunity impacting the booster effect of the second dose [110]. However, a prime and boost approach using two different immunization routes results in enhanced immune responses compared to the group immunized twice with the same route [171]. Low levels of HAd5 vector immunity (below 520 virus-neutralization titer) did not negatively affect the protection efficacy of an HAd5 vector-based influenza vaccine in a mouse model [172]. Conversely, high levels of vector immunity (around 1500 virus-neutralization antibody titer) could be mitigated by either increasing the vaccine dose or using a different vaccination route for booster inoculation. Similarly, the priming of the mouse group with the i.m. route and boosting with the i.n. route using a ChAd-based COVID-19 vaccine expressing broad-spectrum immunogens (modified S and conserved T-cell epitopes) elicited stronger humoral and CMI responses, leading to effective protection against BA.2 compared to other strategies [173] (Figure 4C).

### 6.5. Enhancing Immunity with Diverse Vaccine Platforms

The heterologous prime-boost or mix-and-match approach, involving Ad vectors and other vaccine platforms, has also been employed to enhance the durability of protection and to address fluctuating vaccine supply. A study involving AZD1222 vaccine prime and BioNTech/Pfizer’s BNT162b2 mRNA vaccine boost resulted in an 11.5-fold increase in the anti-S IgG response when compared to a 2.9-fold increase for homologous AZD1222 vaccination [174]. Another study reported a significant boost in S-specific IgG, neutralizing antibodies, and S-specific CD4+ and CD8+ T cells, following AZD1222 prime and BNT162b2 boost [175]. Additionally, AZD1222 (Covishield) prime and Covaxin (inactivated whole virion BBV152) boost elicited superior IgG and neutralizing antibody responses compared to the homologous boost with either vaccine [176].

### 6.6. Enhanced Protection Against Respiratory Pathogens by Mucosal Immunization with Ad Vector-Based Vaccines

Most Ad vector vaccines have been administered via the i.m. route, which primarily induces strong systemic immunity characterized by high circulating antibody levels and T-cell responses. However, because SARS-CoV-2 primarily infects the respiratory tract, i.n. administration has been explored as a promising alternative to enhance mucosal immunity. Unlike the i.m. route, which mainly generates systemic protection, i.n. vaccination stimulates immune responses directly at the site of viral entry. Upon i.n. administration, antigen-presenting cells in the respiratory tract capture the vector or expressed antigen, migrate to local lymph nodes, and activate antigen-specific T cells. This process promotes the formation of tissue-resident memory T cells within the respiratory mucosa, offering long-lasting protection against respiratory pathogens [177]. In addition, mucosal immunization helps induce high levels of mucosal and systemic humoral immune responses. A single i.n. dose of HAd5 vectors expressing either the receptor-binding domain of S (HAd5-RBD) or the complete S ectodomain (HAd5-S) successfully induced high levels of anti-S IgA and IgG in serum and bronchoalveolar lavage, along with potent virus VNAs and strong T cell immunity in transgenic mice (HLA class II-humanized) [178]. These findings support the use of mucosal COVID-19 vaccines and offer potential for preventing SARS-CoV-2 transmission. Likewise, the i.n. delivery of the HAd5-S.Mod vaccine expressing a modified SARS-CoV-2 S protein and the genetic adjuvant, human CXCL9, significantly enhanced airways-specific immune responses compared to i.m. vaccines in mice [179]. This approach not only induced superior humoral and T-cell responses in the respiratory tract but also conferred protection against lethal SARS-CoV-2 infection. The first generation i.m. COVID-19 vaccines have demonstrated efficacy in reducing the disease severity, but they do not protect from infection and virus transmission. However, i.n. COVID-19 vaccines have shown effectiveness in preventing individuals from being infected with SARS-CoV-2 [180].

### 6.7. Ad Vector Modification for Improved Vaccine Efficacy

To reduce the impact of preexisting Ad vector immunity, the hexon hypervariable region (HVR) loops were changed to develop HVR-modified HAd5 vectors [181]. These alterations have also been shown to hinder FX binding [182]. For instance, HAd5 hexon HVR sequences were substituted with the analogous regions of HAd48 to generate a chimeric vector [183], which demonstrated its normal immunogenicity in mice and rhesus monkeys in the presence of high levels of anti-HAd5 VNAs. Likewise, by swapping the HAd5 knob domain with that of HAd3, chimeric Ad vectors showed their improved efficacy to counter preexisting immunity against HAd5 in the mouse model [183]. Additionally, an HAd5 vector incorporating the fiber from BAd4 exhibited reduced innate immune responses and interacted less with blood clotting factors compared to the original HAd5 vector in the mouse model [184]. Inserting peptides into the exposed loops of the Ad fiber or hexon has greatly enhanced the versatility of Ad vectors for targeted delivery and reduced unintended interactions [185,186]. While these modifications have primarily been explored for gene therapy, the approach can be used for targeting specific antigen-presenting cells, thereby boosting immune responses (Figure 4D).

### 6.8. Encapsulation of Ad Vectors to Elude Vector Immunity

The chemical encapsulation of Ad with inert polymeric materials, such as nano and microparticles, liposomes, or minerals, can shield the antigenic surface epitopes from vector-neutralizing antibodies. The shielding of the Ad vector by PEGylation, which involved the covalent attachment of polyethylene glycol (PEG) at specific positions on the capsid, was effective in evading Ad VNAs [187]. The PEGylation of Ad mitigated the innate immune responses and deterred attachment to platelets and other blood factors, thereby averting potential side effects [188]. The shielding of the Ad vector expressing the receptor-binding domain of SARS-CoV-2 S resulted in more than ten times higher SARS-CoV-2-neutralizing antibody titers compared to the non-shielded vectored vaccines due to reduced levels of anti-vector immunity [189]. This vaccine formulation is under investigation in a clinical trial [189].

Biomineralization is another noteworthy approach where the Ad vector is concealed in a calcium phosphate mineral exterior (CaP). A self-biomineralized simian Ad type 23-based SARS-CoV-2 vaccine (SAd23L-nCoV-S-CaP) retained high immunogenicity, and the vector was shielded from the preexisting anti-SAd23L antibodies in vitro and in vivo [190]. The vaccine was thermostable compared to the non-biomineralized counterpart [190].

Both anionic and cationic liposomes have been utilized for encapsulating Ad vectors. The coating of Ad particles with anionic lecithin cholesterol–PEG liposomes ameliorated transfection efficiency and conferred protection from vector VNAs [191]. Likewise, cationic bilamellar dioleoyltrimethylaminopropane/cholesterol (DOTAP:Chol) liposomes’ encapsulation of the Ad vector led to enhanced transduction efficiency in CAR-deficient cells and protected the vector from VNAs in vitro and in vivo [192]. The PEGylation of DOTAP:Chol liposomes not only bolstered transduction efficiency but also reduced cytotoxicity and interactions with blood cells [193]. Ad vectors coated with PEGylated gold nanoparticles (AuNPs) were shielded from anti-hexon VNAs and showed improved transduction in cells independent of CAR and αvβ3 and αvβ5 integrins [194]. The encapsulation of Ad vectors into poly-gamma-glutamic acid–chitosan (γ-PGA-CH) nanoparticles conferred protection from Ad VNAs [195]. Ad vector’s encapsulation into chitosan–bile salt (sodium deoxycholate) microparticles delayed the vector release—a desirable property for vaccination [196]. Furthermore, Ad coated by biodegradable sodium alginate-based microspheres also evaded Ad-specific mucosal and systemic VNAs [197] (Figure 4E).

### 6.9. Use of Bispecific Adapters to Enhance Ad Vector-Mediated Immune Responses

Bispecific adapters are molecules that simultaneously bind to two different targets. These adapters are used to bridge interactions between viral vectors, therapeutic molecules, or cell surface receptors (Figure 4F). The adapter molecule CFm40L efficiently facilitated Ad-mediated gene delivery to dendritic cells (DCs), thereby proving crucial in enhancing immune responses [198]. Furthermore, CFm40L-targeted HAd5 induced DCs maturation and the upregulation of IL-12 expression, leading to enhanced immunogenicity [198].

### 6.10. Use of Signaling Molecules or Peptides to Increase Ad Vector-Mediated Immune Responses

Altering the cellular trafficking of the desired immunogen by including specific domains or peptides can significantly impact the levels of antigen-specific immune responses. An autophagy-inducing peptide C5 (AIP-C5) of *Mycobacterium tuberculosis* helps in autophagy-mediated antigen processing in lysosomes, leading to an alternative route of MHC-I-dependent and MHC-II-dependent antigen presentation [199] (Figure 4G). The i.n. immunization of mice or golden Syrian hamsters with the HAd5 vector expressing SARS-CoV-2 S and AIP-C5 displayed comparable S-specific humoral responses but significantly elevated levels of S-specific CMI responses compared to the groups vaccinated with the HAd5 vector expressing only SARS-CoV-2 S [200]. Similarly, the immunization of mice with the HAd5 vector expressing the influenza virus nucleoprotein (NP) with AIP-C5 elicited significantly higher levels of NP-specific CD8+ T cell responses compared to the groups vaccinated with the HAd5 vector expressing only NP [201]. These results indicate that including AIP-C5 in the vaccine formulation enhanced the antigen-specific CMI responses against the immunogen of choice.

Signaling peptides or small molecules (~20–30 amino acids) control a specific signaling pathway, its secretion, and post-translational modifications [202]. The inclusion of 20 amino acid (METDTLLLWVLLLWVPGSTG) signal peptides derived from the murine IgG kappa chain, upstream of the transgene in an SAd23 vectored malaria vaccine, augmented antibody avidity and increased IL-2 and IFN-γ production by CD4+ T cells [203]. A TLR signaling molecule, a TRIF-related adaptor molecule (TRAM), when inserted into the HAd5 or ChAd63 vector, led to the increased production of IL-8 in HeLa cells [204]. Mice immunized intradermally (i.d.) or i.m. with the HAd5 vector co-expressing TRAM with the transgene of interest bolstered CD8+ T cell responses with a minimal increase in CD4+ T cell responses [205].

Another interesting concept is to include a short peptide in the gene’s open reading frame to help boost the expression level of the transgene of interest. Various peptide tags can stabilize mRNA, thereby increasing its half-life or preventing protein degradation [198]. A 21-nucleotide-long cis-regulatory motif (Exin21; CAACCGCGGTTCGCGGCCGCT), which encodes a heptapeptide (QPRFAAA), led to significant increases in the expression levels of SARS-CoV-2 E, S, or the M protein when inserted in frame of the transgene [206]. Furthermore, Exin21 also boosted the expression level of anti-SARS-CoV monoclonal antibodies and the SARS-CoV-2 S-based mRNA vaccine [199]. Other such tags, like a 13-amino-acid-long STABILION (KDGKKDKKEEDKK) from the *Drosophila* 26S proteasome and a 16-amino-acid-long peptide from transcription factor DP-1 (EDDEEDDDFNENDEDD) have also been reported to boost the expression of the transgene [207]. However, this approach has not been employed with Ad-based vaccines. The inclusion of these peptide tags along with the transgene may help boost its expression, thereby increasing immunogenicity and reducing the vaccine dose.

## 7. Conclusions and Future Directions

Ad vector-based vaccines have played a pivotal role in controlling the COVID-19 pandemic, demonstrating strong clinical efficacy and marking a significant milestone in vaccine development. Their advantages, including rapid scalability, the robust induction of cellular immunity, and established vector technology, make them promising candidates for a broad range of infectious diseases. This review also highlights the preclinical and clinical efficacy of these vaccines, emphasizing their effectiveness in eliciting strong immune responses. However, further optimization is essential to enhance their immunogenicity and address certain limitations. Strategies such as targeting additional SARS-CoV-2 immunogens beyond the S protein, developing bivalent or multivalent S-based vaccines, and utilizing less prevalent human or nonhuman adenoviral vectors could improve immune coverage and durability. Moreover, modifying immunization strategies, including heterologous prime-boost regimens and mucosal delivery, may enhance protection, particularly against respiratory pathogens. Enhancing vector design through modifications, such as encapsulation to evade preexisting immunity, the use of bispecific adapters, and the incorporation of signaling molecules or peptides, could further refine vaccine efficacy. While these approaches offer promising avenues for improvement, future research must focus on achieving broad and durable protection against emerging variants while minimizing side effects. Additionally, implementing targeted vaccination programs for significant animal reservoirs, such as mink and deer, could play a crucial role in preventing zoonotic spillover and limiting the emergence of novel variants—reinforcing global pandemic preparedness.

## Data Availability

Not applicable.
